# Universal Codons with Enrichment from GC to AU Nucleotide Composition Reveal a Chronological Assignment from Early to Late Along with LUCA Formation

**DOI:** 10.3390/life10060081

**Published:** 2020-06-05

**Authors:** Anastas Gospodinov, Dimiter Kunnev

**Affiliations:** 1 Roumen Tsanev Institute of Molecular Biology, Bulgarian Academy of Sciences, Acad. G. Bonchev Str. 21, Sofia 1113, Bulgaria; agg@bio21.bas.bg; 2 Department of Molecular & Cellular Biology, Roswell Park Cancer Institute, Buffalo, NY 14263, USA

**Keywords:** origin of life, Darwinian evolution, universal genetic code, LUCA, pre-LUCA, first codon, RNA–peptide world, aminoacyl-tRNA synthetases, aaRS, bridge peptide, GC-rich codons, first life, first organism

## Abstract

The emergence of a primitive genetic code should be considered the most essential event during the origin of life. Almost a complete set of codons (as we know them) should have been established relatively early during the evolution of the last universal common ancestor (LUCA) from which all known organisms descended. Many hypotheses have been proposed to explain the driving forces and chronology of the evolution of the genetic code; however, none is commonly accepted. In the current paper, we explore the features of the genetic code that, in our view, reflect the mechanism and the chronological order of the origin of the genetic code. Our hypothesis postulates that the primordial RNA was mostly GC-rich, and this bias was reflected in the order of amino acid codon assignment. If we arrange the codons and their corresponding amino acids from GC-rich to AU-rich, we find that: 1. The amino acids encoded by GC-rich codons (Ala, Gly, Arg, and Pro) are those that contribute the most to the interactions with RNA (if incorporated into short peptides). 2. This order correlates with the addition of novel functions necessary for the evolution from simple to longer folded peptides. 3. The overlay of aminoacyl-tRNA synthetases (aaRS) to the amino acid order produces a distinctive zonal distribution for class I and class II suggesting an interdependent origin. These correlations could be explained by the active role of the bridge peptide (BP), which we proposed earlier in the evolution of the genetic code.

## 1. Introduction

Several theories have sought to explain the establishment of the genetic code and the forces behind it [1]. Francis Crick’s frozen accident model [2] was one of the first attempts for such an explanation, postulating that the codon selected for a particular amino acid is initially set by chance, and could not be changed later as multiple important functions would depend on it. Therefore, if theoretically life could start all over again, under the same conditions, the genetic code could be quite different. This model, however, does not explain why some codons could be grouped by the physicochemical properties of their cognate amino acids. Approximately at the same time, the stereochemical theory tried to elucidate the specificity between amino acids and their cognate codons [3,4,5]. The stereochemical theory postulates that the physicochemical properties of a given amino acid facilitate its codon recognition, and this relation is the basis for establishing the correspondence between an amino acid and its future tRNA. Approximately ten years ago, the chemical principles governing interactions of specific RNA with amino acids were investigated in detail. The authors found that, indeed, for certain amino acids, their aptamer interactions could explain the selection of their cognate codon [6]. Given that tRNA-amino acid recognition is currently performed by a completely different mechanism, it seems unlikely that the stereochemical properties could have been the driving force leading to the establishment of the genetic code.

Higgs and Pudritz proposed that the order of amino acid encoding reflected their prebiotic abundance [7]. They divided the amino acids into an early group glycine (Gly), alanine (Ala), aspartate (Asp), glutamate (Glu), valine (Val), serine (Ser), isoleucine (Ile), leucine (Leu), proline (Pro), and threonine (Thr) and a late group which includes the remaining amino acids that were much less abundant or even absent in prebiotic conditions. Late amino acids started to be utilized after the metabolism became capable of producing them. In a way similar to Higgs, Bernhardt and Patrick considered that the simplest and most abundant prebiotic amino acid Gly, and other small hydrophilic amino acids, were the first to become coded and have GC-rich codons [8]. Arg is also coded by GC-rich codons, but the authors omitted it from the earliest encoded amino acids since most (but not all) simulation experiments suggested that Arg was not present under prebiotic conditions [8]. The coevolution hypothesis of the genetic code proposed that the encoding of new amino acids was determined by the evolution of their synthetic pathways within the primordial biochemical system [1,9,10]. While this hypothesis may be valid for certain late amino acids, it is unlikely to be correct for the earliest of them, as this would demand complex protein-based enzymatic activities. In our view, the synthetic pathways and metabolism evolved to achieve “independence” from the environmental supply of ingredients and energy, and this certainly would have been an extraordinarily powerful selection force.

By applying 60 different criteria, Trifonov created a chronological order of incorporation of amino acids into the newly formed living structures [11]. These criteria are based on a variety of hypotheses for codon formation, phylogenetic analyses, meteorite amino acids findings, and combinations of many single-factor and multi-factor criteria. Here is Trifonov’s order from early to late: Gly, Ala, Asp, Val, Pro, Ser, Glu (Leu, Thr), Arg, (Ile, Gln, Asn), His, Lys, Cys, Phe, Tyr, Met, and Trp where in parentheses are amino acids coded within the same timeframe [11]. This list largely resembles the abundance of the amino acids produced from the famous Miller experiment [12] and mostly follows the early/late amino acids list based on their prebiotic abundance [7]. In Trifonov’s order, Gly and Ala are the first two; Pro occupies the fifth position after Asp and Val, and arginine (Arg) is in tenth place, which according to our concept should be different.

In another hypothesis, de Farias and coworkers agree with Higgs and Pudritz that some of the most abundant amino acids (Ala, Asp, Val and, Glu) were the first to become encoded [13]. The driving force for codon assignment in their model is the correlation between (dinucleotide) codon hydropathy and amino acid hydropathy [13,14,15]. The authors proposed a mechanism where the codon/anticodon hybridization of proto-tRNAs led to the formation of dimers and small peptides. The newly formed peptides could stabilize RNA, and the surviving proto-tRNA oligonucleotides would give rise to the peptidyl transferase center (PTC) of rRNA and mRNA. The order of amino acid encoding follows a coevolution model in which the simple amino acids were encoded before the more complicated amino acids. In their sequence of events, Arg is needed much later, together with the other more complex amino acids.

The initial formation of peptides by codon/anticodon proto-tRNA hybridization proposed by de Farias and coworkers seems quite likely. A similar (but not identical) mechanism for the formation of earliest peptides (hybridization dependent peptides HDPs) was proposed by the authors of this article [16]. In contrast to the stereochemical concept and codon/amino acid hydropathy, we have recently proposed the “bridge peptide” (BP) hypothesis to explain the primordial recognition of a specific RNA sequence and its amino acid [16]. While we recognize the importance of amino acid abundance in the stages of evolution preceding specific aminoacylation (we call it “chemical code” in Table 1), we emphasize the contribution of a newly coded amino acid, based on its physicochemical properties towards the perpetuation of the primeval RNA–peptide complexes. Thus, while most hypotheses regarding the establishment of the genetic code assume implicitly that prebiotic conditions were “set” to favor the stability of the early living entities (i.e., the amino acid abundance or various forms of chemical fit between nucleic and amino acids favoring the establishment of the genetic code and, consequently, stability), we postulate that from the very beginning, the formation of a genetic code was driven by Darwinian selection [16]. This started with the establishment of a primitive translation by BP-driven RNA–amino acids interactions. We postulate that the codon sequence did not influence amino acid selection. In our view, the coding sequence of a particular amino acid should have been a direct result of two sets of circumstances: (1) the available sequences at the time of codon assignment and (2) the selection process directed by the physicochemical properties that the (newly coded) amino acid contributed to the perpetuation and survival of the RNA–peptide living complex, i.e., its functional importance. Before going into details on the GC/AU content of codons, we need to illustrate our expectations for the amino acid abundance and the nucleotide composition in the prebiotic environment.

## 2. Prebiotic Amino Acid Abundance

In 2008, De Santana and coauthors [17] summarized most of the simulation experiments and observations seeking to answer what was the most probable amino acid composition on prebiotic Earth. The authors considered both exogenous (amino acids synthesized outside Earth and delivered to our planet by interplanetary dust particles, meteorites, comets, etc.) and endogenous sources synthesized on Earth in atmospheric mixtures, hydrothermal vents, or using different energy sources such as UV radiation, electric discharge, cosmic rays, and meteor impacts. We use these data (Table 1) as a plausible estimate of prebiotic amino acid abundance.

In addition to the canonical amino acids, it is shown that in simulated experiments, many different types of species are synthesized. We can be sure that a variety of combinations and interactions occurred with many modified forms of amino acids in the first life complexes [18]. However, once Darwinian evolution took place, the selection forces allowed the emergence of the 20 amino acid canonical set (and some noncanonical) which occupy “chemistry space” large enough for life to prosper [19]. Ilardo and coauthors compared the encoded amino acid alphabet to random sets of amino acids (from a computationally generated compound library containing 1913 alternative amino acids that lie within the same molecular weight range) in terms of relevant physicochemical properties. Sets that cover the “chemistry space” better were extremely rare and energetically costly, indicating that the 20 amino acids found within the standard genetic code are the result of considerable natural selection. The authors also suggest that the canonical set represents a global optimum, such that any aqueous biochemistry would use a very similar set [19].

## 3. Prebiotic Nucleotide Composition of RNA

We assume that archaic evolutionarily conserved RNAs are the best estimate we have for the composition of early RNAs. Multiple studies indicate that the most archaic RNAs are GC-rich. It is reasonable to accept that RNAs involved in translation are highly conserved [20]. Specifically, in tRNA, the sequences in the acceptor stem and anticodon loop are considered the most ancient; in rRNA, these are helix 44 [21] of the small subunit (SSU) and the PTC center [22] of the large subunit (LSU). Burton and coauthors used a statistical permutation test of highly homologous tRNA sequences to suggest a model of tRNA origin. According to this model, the first primordial tRNA sequences are truncated contiguous (GCG)_3_ repeats from which the acceptor stem formed and (TAGCC)_4_ from which the D-loop formed. The anticodon (Ac) and T loops originated from a 17nt microhelix sequence noncontiguous repeat (~CCGGGTTCAAAACCCGG)_2,_ which in turn, most likely originated from simpler repeats. The middle sequence “TTCAAAA” is the U-turn, which may have formed just a step later [23,24]. Recently, Thompson and coauthors analyzed rRNA from 133 representative organisms [25] looking for primordial motifs containing circular X codes (i.e., codes which are in transition between the primitive nonspecific codes and the modern genetic code). In this study, uX motif-a (the closest to h44) is ACACCGCCC; uX motif-B, and motif-D (which are part of the PTC) are aCcTCGATGTCGGCT and GtGAGCTGGGTTt respectively, and are, indeed, GC-richer [25]. Here, the capital letters represent universally conservative nucleotides, and the small ones represent less conserved nucleotides. These studies indicate that the most ancient sequences of tRNA and rRNA are GC-biased, supporting the notion that RNA oligonucleotides in the RNA–peptide complexes that initiated life were also GC-rich.

Aside from molecular relics, there are thermodynamic, environmental, and replicative reasons for the GC-richness of primordial RNA. GC base-pairing established by three hydrogen bonds is thermodynamically more stable than AU base-pairing with two hydrogen bonds. This suggests that complexes containing GC-rich oligonucleotides would be more stable and likely persisted longer in the environment.

UV irradiation, abundant on prebiotic Earth, causes the formation of cyclobutane type dimers and uridine–uridine adducts in frozen and thin-film water samples [26]. Although the role of UV in the prebiotic chemistry depended on the wavelengths and was very important for the synthesis of nucleotides [27], it is still conceivable that, at the level of nucleotide polymer (RNA) in the mostly surface-related environment of primitive RNA–protein complexes, the content of AU would have been actively diminished relative to GC pairs.

Experimental attempts to achieve in vitro nonenzymatic RNA replication also suggest the GC-richness of the initial RNA complex. In these experiments, researchers quickly realized that the process of nonenzymatic RNA replication is significantly GC-biased as the addition of either A or U (or both) inhibited the polymerization reaction [28]. Further experiments succeeded in achieving polymerization with four nucleotides on immobilized RNA, but the primer extension with adenosine and uridine had a lower rate [29]. An improvement in incorporation has been achieved by 2-methylimidazole-activated nucleotides and activated “helper” oligonucleotides in which A and U were incorporated into RNA as part of an oligonucleotide (at least a trimer) [30]. The efforts (to accomplish four nucleotides nonenzymatic RNA replication) were driven by the need to show that prebiotic replication could occur faster than the degradation of functional ribozymes: “a must” in RNA world theory. This, however, requires a relatively efficient replication process (with four nucleotides), producing relatively long and complicated ribozymes to serve as a selection pool for an RNA replication ribozyme. In the RNA–peptide hypothesis, high fidelity nonenzymatic RNA replication using all four nucleotides is not essential. The process of nonenzymatic RNA synthesis would still be important, but only to maintain the pool of oligonucleotides irrespective of sequence, some of which would survive longer. After the initiation of Darwinian evolution of the RNA–peptide complexes, an RNA replication peptide (RRP) could emerge, and then A and U would start to be incorporated more frequently, creating the RNA “sequence landscape” necessary for more variations and extra codons [16].

As a result of the prebiotic conditions, the pre-synthesized pool of amino acids and short RNAs would allow interactions in all possible ways in “single pot” or in “step by step” reactions. We assume that the prebiotic synthetic environment supplied amino acids and short RNA for a time long enough until Darwinian evolution emerged. 

## 4. Order of Codon Establishment—GC-Rich Were Early and AU Late

If indeed, the initial RNAs were predominantly GC-rich, so would be the codons for the first coded amino acids, with AU-rich codons appearing later in the evolution of the genetic code. On the other hand, clearly, nucleotide content cannot be the driving force of codon composition and selection. We assume that the factor determining the time of encoding of a particular amino acid could only be its physicochemical properties related to the survival of the living structures. These would confer specific properties increasing the RNA–peptide complex stability as a network of more adaptive interactions and would be selected when the carrier amino acid is included in the code. The order of codons then should parallel the evolution from short peptides to longer motif-containing peptides to folded proteins forming catalytic centers [31]. The first amino acids to become coded should have facilitated the stabilizing function of short peptides, the coded next “middle” amino acids should have been important for folding and the formation of tertiary protein structures, and finally, the AU group should have become necessary for fully developed proteins with a fixed start and end. 

**Figure 1:**
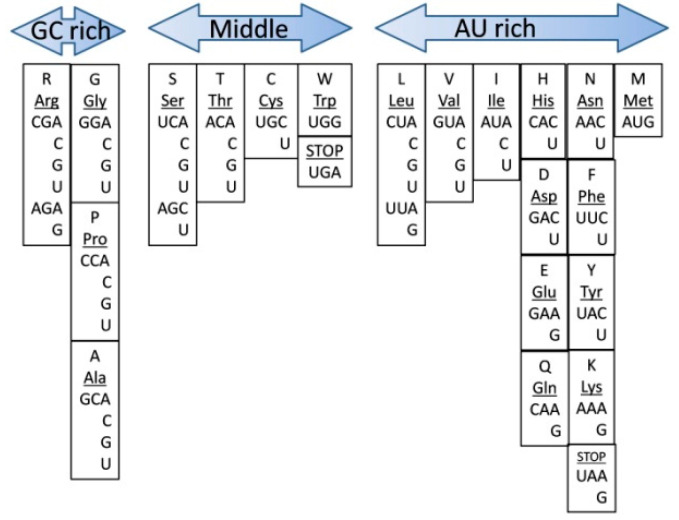
Arrangement of the canonical amino acid codons by their GC-richness. Every amino acid and its cognate codon(s) are placed from left to right according to two criteria: (1) The codon is placed more to the left if its first and middle bases are G or C with the middle base taking precedence over the first 5′ base. (2) The number of the bases in the third (wobble) place. The more bases (codons) are there, the earlier the amino acid was encoded (positioned to the left). The first criterion has priority; the second is used to move the amino acid within the group established by the first criterion. Codons with the same GC content are placed vertically.

What is the proper way to order the codons (and their corresponding amino acids) based on their nucleotide content? We hold that the following rules governed the establishment of the code:

1. The middle base and the first 5′ base should have had maximum contribution to codon formation since those do not vary a lot in the codons for a particular amino acid and are the most important for codon–anticodon recognition. In this rule, however, the second nucleotide has priority over the first nucleotide in the 5′ position. 2. The third base (wobble base) is the most variable. Consequently, the more bases there are in the third position for the same amino acid, the less other amino acid(s) competed functionally to take this codon. Following this logic, an amino acid with more bases in the third position should be considered an “earlier” one. According to our model, this arrangement should represent the relative time of the assignment of an amino acid to its cognate codons on a global scale. Amino acids with the same “score” for GC-richness are positioned vertically, indicating the same period of origin. There appear three major relative groups, based on their GC content (Figure 1). The “GC rich” group includes arginine (Arg), glycine (Gly), proline (Pro) and alanine (Ala). The “middle” group consists of serine (Ser), threonine (Thr), cysteine (Cys), tryptophan (Trp) and the UGA stop codon. In the “AU rich” group are leucine (Leu), valine (Val), isoleucine (Ile), histidine (His), aspartic acid (Asp), glutamic acid (Glu), glutamine (Gln), asparagine (Asn), phenylalanine (Phe), tyrosine (Tyr), lysine (Lys) and methionine (Met) (also coding the initiation of protein translation), UAA and UAG stop codons.

The columns with the same GC content suggest that the chronological order based on GC-richness is valid only on a global scale, and the boundaries between the groups are relatively subjective. Based on the GC criteria only, within a group or column, it is unreasonable to tell which amino acid was assigned first. Therefore, on top of the general rule (GC-rich early, and AU-rich late), there are more fine processes that determine the “need” for a specific amino acid and its chronological incorporation into the genetic code. It is also possible for the codon assignment within a group (with the same GC-richness) to be a combination of the “need” for a particular amino acid and Francis Crick’s frozen accident model. 

If we take criteria N6′—“Stability of complementary interactions” from Trifonov’s chronological analyses [11], which is more relevant to Darwinian survival and evolution, we see the following order: Ala, Gly, Ser, Pro, Arg, Asp, Thr, Cys, Glu, (Val, Trp), His, Leu, (Met, Gln), Ile, Asn, Tyr, Phe, and Lys. This list remarkably resembles the list we propose (Figure 1), arranged only by ordering codons according to their GC content. “Stability of complementary interactions” reflects the thermostability of nucleic acids as part of triplets: first for codon–anticodon interactions where the melting enthalpies of the dinucleotide stacks were measured; second, DNA stability of the triplet pairs where ionic-strength dependence of melting profiles was measured; and third, the same type of measurement, but for duplex RNA genes [11]. In those assessments, GC-rich codons show a higher level of thermostability. The first five amino acids Ala, Gly, Ser, Pro, and Arg are the same as in Figure 1. Ser in our list comes immediately after the GC-group, and it possesses the most GC-rich codons in the middle group. The last amino acids, according to Trifonov’s N6′ criterion also correlate well with our AU-rich group, where Met, Gln, Ile, Asn, Tyr, Phe, and Lys are all in the same group. This remarkable correlation suggests that the GC-rich codons with more thermodynamically stable RNA structures and their associated amino acids would be those that confer the highest stability to the RNA–peptide complex and not the most abundant amino acids.

## 5. Codon Groups

The distribution of the codons and their cognate amino acids in Figure 1 is conditionally separated into three groups: GC-rich group, the middle group, and AU-rich group. The GC and the middle group have G or C nucleotides in the second place in the codon. In the middle group, the first (5′) base becomes A or U. In the AU-rich group, the nucleotide in the second place of the codon is A or U. We need to describe the importance of each group for protein evolution; from short peptides toward longer motifs and developed proteins with a fixed start and end.

### 5.1. GC Group

Gly and Ala are the most abundant and the simplest amino acids synthesized from prebiotic chemistry (Table 1). While the codons for Gly and Ala are GC-rich, GC-richness does not appear to be linked with amino acid abundance in the prebiotic environment. Thus, relatively abundant amino acids like Leu, Ile, Val, Asp, Glu, and Thr have either GC- or AU-rich codons. Therefore, we can safely assume that the most abundant, Ala and Gly, were among the first to become coded not due to their abundance, but due to their properties. What were the properties of Gly and Ala that led to their early selection, and how did they contribute to Darwinian evolution? The lack of charge (and lack of RNA interaction) makes them perfect “spacers” that do not interfere with the properties of the other amino acids and ions. Furthermore, these two amino acids have a high conformational flexibility to the peptide bond, allowing any other charged amino acid to adapt its position within the same peptide. The ability of peptides to adjust their size and conformation would have had a dramatic impact on the stability of the complex and, therefore, they would be selected for this. This could be extremely important in the earliest RNA–peptide complexes where the primordial RNA size and shape were not completely stabilized. The next in GC-rich group are Pro (codons CCN) and Arg (codons CGN, AGG and AGA) all with C or G in the middle base. Pro is a nonpolar neutral amino acid, included in the list of the ten earliest amino acids [7]. Pro abundance in simulations is not as high as Gly or Ala and is most likely about 10 to 20 times lower than Gly (Table 1) [17]. Pro destabilizes alpha-helix structures and makes “kinks,” which can be useful in forming tight turns and in protein folding by changing the free energy of folding [32]. In short peptides, Pro makes kinks, thus forming different shapes, which might have been particularly suitable for fitting peptides with the 3D structure of RNA. Pro has the ability to generate kinks in the peptide, irrespective of its length. Due to this unique ability, Pro is likely the very first determinant of the 3D structure in peptides and should have played a vital role in early hybridization dependent peptides (HDPs), described before [16], by contributing to the stability of the RNA–peptide complex. The role of HDPs will be discussed later in the text.

Some authors have excluded Arg from the earliest amino acids involved in codon formation, and it is considered a late amino acid [7,8]. On the contrary, Griffith has suggested that Arg should be the first coded amino acid due to its ability to interact with RNA and to facilitate nucleic acid replication [33]. We also consider Arg as one of the first coded amino acids. In our hypothesis, Arg is an essential part of RNA-binding properties of BPs, with BPs having stabilizing function in the initial RNA–peptide complex. Being positively charged in a wide pH range (pI = 10.76), Arg is the best canonical amino acid which interacts with RNA. A recent study by Chen and coauthors showed that Arg must be an essential part of any RNA-interacting primordial peptide [34]. The interaction of Arg with aptamer RNAs was shown in several studies [6]. In a paper reporting evidence for a stereochemical hypothesis in the genetic code formation, Yarus and coauthors described multiple Arg binding sites on RNA, suggesting that these are consistent with multiple opportunities for RNA–amino acid interaction [6]. These data are in line with the results obtained by Tao and Frankel [35] and by Famulok and coworkers [36] who showed preferential binding of Arg to aptamers with sequences biased toward the GC-rich sequence [35,36]. In addition, Arg-rich peptides and the peptides containing the arginine-rich motif (ARM) are part of certain viral proteins interacting specifically with GC-rich RNA [37]. ARM containing proteins have been shown to bind RNA with sequence specificity. For example, ARMs of phage l bind their own boxB RNA 16-fold better than the related phage P22 boxB RNA [38]. The Rev-binding element (RBE) and anti-Rev aptamers have a 10-fold higher affinity for certain Rev ARM variants differing by a single amino acid [35,38]. In addition, a study of the hydrogen bonds of nucleic acid bases and the amino acids demonstrate the preferential interaction for Arg and Lys with guanine [39]. 

The authors showed some clear preferences for particular pairings of amino acids and bases. Thus, Arg and Lys strongly favor guanine (Pcomb < 0.0001, Roe = 24.5 and 4.2) and largely account for the abundance of hydrogen-bond interactions with this base. To a lesser extent, Asp and Gln prefer adenine (Pcomb < 0.0001, Roe = 3.0 and 4.2). The combinations are by no means exclusive, and these amino acids also interact with other base types, albeit less often. For example, arginine also makes a larger than expected number of interactions with thymine and adenine (Pcomb < 0.001 and 0.0001) [39].

Together, these data demonstrate the ability of Arg-rich motifs to perform sequence-specific binding to various sequences as part of relatively short motifs. We conclude that Arg is biased to bind GC-rich sequences due to a combination of its positive charge and hydrogen-bond interactions, and its binding could have some degree of sequence specificity as part of short peptides. We postulate that these properties of Arg in peptides (not in its free form) would have been critical for the binding of the BP with specific GC-rich RNA sequences/structures. In addition to these Arg–peptide–RNA interactions, the positively charged guanidinium group can interact with negatively charged phospholipids, which may have been present into the prebiotic milieu. Thus, Arg, as part of a peptide, would facilitate the formation of a lipid complex through combined electrostatic forces and facilitate lipid compartmentalization and stability [40]. 

While the properties of Arg make it extremely valuable in any RNA–peptide interactions, the main argument against it being an early amino acid is centered on the assumption of its absence from the prebiotic environment [7,8,41,42]. There is, however, evidence suggesting that Arg could and should be synthesized under prebiotic conditions. The prebiotic mixture contained formaldehyde, hydroxylamine, ammonia, hydrogen cyanide, formamide, diaminomaleonitrile, etc., which [43,44,45,46,47,48,49] could have permitted the synthesis of Arg. Some of these components (e.g., formamide, hydrogen cyanide) are also suitable precursors of nucleotides [46,48,49,50]. In a recent review, Sasselov, Grotzinger, and Sutherland described the prebiotic chemistry for the synthesis of nucleotides, amino acids, and lipids. Depending on the average N and O oxidation, different nucleotides, lipids, and amino acids could be synthesized, including Arg [51]. 

Due to the properties of Pro (making kinks) and Arg (binding GC-rich RNA), these amino acids would have been greatly beneficial to stabilize the interactions of RNA and primitive peptides. At the same time, Gly and Ala, while having no direct effect on RNA–peptide interactions, likely provided the necessary spacing between Arg and Pro to spatially better fit and adapted HDPs to RNAs 3D structure.

### 5.2. “Middle” Group

We expect that amino acids critical for protein folding to belong to the middle group. Salt bridging and hydrogen bond formation are essential for protein folding. These are the main physicochemical properties by which these amino acids assist protein folding. The amino acids important for the formation of hydrogen bonds and salt bridges are part of the “Middle” and “AU-rich” groups e.g., Ser, Thr, Tyr, Asp, Lys, Glu, and Asn. The codon for Ser, with C or G middle base, is remarkably close to the GC-rich group, so we would expect that it acquired its function earlier than the rest of the “middle” group. Furthermore, it seems possible that the Ser codon originated from the GC group by replacing the CCN or GCN part of Pro or Ala with UCN. Thr, Cys, Trp, and UGA stop codons have a G or C middle base but start to incorporate A or U in their first or third base positions since the middle G or C were already occupied by the GC group. Cys possesses a sulfhydryl (SH) group, and it can form disulfide bonds or be present in protease catalytic centers. It is logical to assume that the selection of Cys occurred only after the peptides became long enough to fold, and disulfide bonds began to play a role in keeping the tertiary structure intact, or the peptides began to have protease catalytic centers. In prebiotic synthesis, UV light drives the formation of iron–sulfur clusters, and these can be stabilized by cysteine-containing peptides [52]. The cysteine-rich peptide complexes could be involved in an iron–sulfur cluster-dependent metabolism, facilitating protocell formation [52]. These primitive reactions may be harnessed by the first protocell; however, they will not contribute to its survival until genetically based Darwinian evolution is established. In that case, as long as we are looking for codon assignment, the role of Cys should be examined as part of coded folded motifs.

### 5.3. AU-Rich Group

Leu, Ile, Val with U as middle codon base are hydrophobic nonpolar aliphatic amino acids. The hydrophobic nature of these amino acids facilitates lipid interactions today, and they are vital parts of transmembrane domains. The evolutionary development of the initial RNA–peptide complex would have led to the functional integration of membranes and, thus, better compartmentalization and controlled interactions with the environment. Leu, Ile, Val functions are not limited to membrane interactions but are also crucial for maintaining hydrogen bond dependent interactions, which require more advanced folding motifs and much longer peptides than the earliest oligomers. For example, the hnRNP K homology (KH) domain consisting of three α-helices packed against a three-stranded β-sheet contains a conservative pattern (I/L/V)IGxxGxx(I/L/V) capable of interacting with single-stranded nucleic acids [53,54]. Another example is the adenosine phosphate-binding domain of class I aminoacyl-tRNA synthetases that possess backbone brackets at position 1361 with conservative Leu, Val, or Ile [55]. These show the three amino acids are important for the maintenance of certain folded structures, therefore it should be assigned to a codon after short peptides were established. Phe, His, Asn, Tyr, Gln, and Glu are important for primordial structural motifs that feature protein folding such as α- and β-hairpins, β-meanders, β-α-β elements. Asp is a relatively abundant amino acid and might have played a significant role as an Mg^2+^ or Fe^2+^ or Mn^2+^ holder in advanced catalytic centers. In that case, the Asp codon assignment might have become necessary later when more advanced modified motifs were developed. It is quite possible also that Asp might have been incorporated by chance in short peptides much earlier in evolution without codon assignment, due to its abundance. Met is another important amino acid containing sulfur with a linked methyl group. Met is also used as a donor by several methyltransferases for a variety of functions, but all of these require a high level of enzymatic activity.

It has been shown experimentally that some of the aromatic and charged amino acids from the AU group are important for protein condensate phase separation. A study of the Fused in Sarcoma (FUS) protein family (a class of intrinsically disordered scaffold proteins) by Hyman and coworkers has shown that the amino acids that determine phase separation are Arg and Tyr [56]. This requires Arg and Tyr to physically interact to promote separation from the surrounding environment. Considering the fact that this function does not include RNA binding features, we hypothesize that Tyr–Arg and peptide–peptide interactions could have become important at a later stage when the living complex begins to separate and becomes an individual protocell. 

Both Lys and Arg are basic with positively charged residues, and both interact with RNA. Why then, would Lys (assigned to AU-rich codons AAA and AAG) be coded late, while Arg should be coded first, as both have a similar physicochemical nature? A reasonable explanation could be the stronger interaction of Arg, which is better suited to hold to a relatively small RNA–peptide complex. An example of how small differences in properties drives different biological outcomes is the phase separation phenomenon by Arg and Tyr interactions. If Arg is replaced by a Lys residue, the phase separation is dramatically reduced [56]. Lys would be important later in evolution for better functional “tuning” when the peptides become long enough to include different motifs. Thus, from an evolutionary point of view, Lys was not important until the last period of the genetic code formation, since Arg covered most of the needs for a basic positively charged amino acid.

The start codon AUG for Met and stop codons UAA and UAG (or any stop codon) should have been established after the assignment of several coded amino acids would have allowed for long enough coded peptides. Initially, short peptides would have functioned solely dependent on the physicochemical properties of their constituent amino acids. Frequent misincorporation into a simple peptide would not have had a significant effect on its function, since the “correct” amino acids would be sufficient for its function. Its activity would have depended on the physicochemical features of its constituent amino acids rather than on its tertiary structure. In short peptides, the fixed start and stop were likely unimportant. As evolution advanced, the peptides became longer, folded, and their activity started to depend on catalytic centers (e.g., formed motifs). These should have required fixed start and fixed end and “in-frame” translation to preserve the correct amino acid sequence.

## 6. GC/AU Zonal Distribution of aaRS Classes

Our hypothesis posits that the codon assignment happened from GC-rich toward AU-rich codons (Figure 1, from left to right). We illustrate a correlation between the GC-richness of codons and the evolution of the proteins from short peptides to long folded proteins. If these are correct in agreement with the RNA–peptide concept for the origin of life, the evolution and the phylogenetic tree of the aminoacyl-tRNA synthetases (aaRSs) should also correlate with the proposed order of codon assignment. Therefore, the investigation of the phylogenetic tree of aaRSs should be a suitable test for the validity of our hypothesis. At the same time, in considering the evolutionary relations of aaRSs, we hold that codon assignment of specific amino acids did not have to require fully developed aaRSs. Therefore, the order of codon assignment (Figure 1) reflects coevolution with the initial proto-aaRSs, not later evolutionary adaptations on the path to modern aaRSs. Moreover, during the last universal common ancestor (LUCA) period, horizontal transfer of genes (HGT) was a common phenomenon, and peptide motifs may have “traveled” in any direction, complicating phylogenetic analysis [1,57,58].

Aminoacyl-tRNA synthetases (aaRSs) are certainly among the most ancient proteins, however, because of the need to recognize all 20 canonical (and some noncanonical) amino acids and all codons, these proteins evolved and diversified dramatically. As a result, it is difficult to trace any specific sequence or motif(s) back to its initial form. There are two classes of aaRS—class I aaRSs attach the amino acid to the 2′-OH of a terminal adenosine nucleotide on tRNA, while class II aminoacylate the 3′-OH ribose group. The class I aaRSs specify eleven amino acids (Met, Val, Ile, Leu, Cys, Glu, Gln, Lys, Arg, Trp, and Tyr) and class II specify ten amino acids (Ala, His, Pro, Thr, Ser, Gly, Phe, Asp, Asn, and Lys) [59,60]. Lys can be charged by aaRSs of both classes. AaRS class I and class II have evolved independently, and their catalytic mechanisms are different.

**Figure 2:**
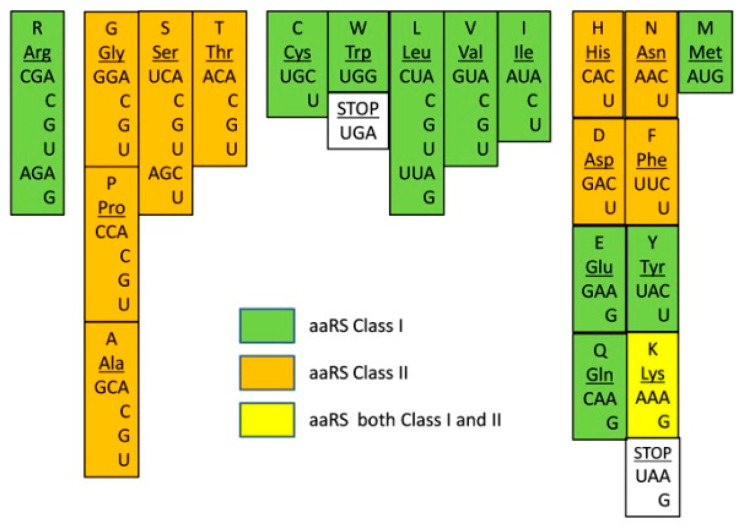
Zonal distribution of class I and class II aminoacyl-tRNA synthetases (aaRS) in relation to codon GC-richness. A modified Figure 1 is presented in which every amino acid is color coded based on the class of the aaRS that charges it—class I, class II or both. Note that the zonal distribution appears by overlaying of the corresponding class aaRS over the order of codons in Figure 1 without additional adjustments.

In order to investigate the correlation between the GC-richness and aaRSs evolution, we decided to overlay the corresponding aaRS classes for each amino acid over the codon assignment order. After the overlay, we observed a peculiar zonal or periodic distribution appeared naturally (Figure 2). All GC-rich codons carry amino acids associated with class II aaRS (Gly, Ala, Pro) except for Arg. The left side of the middle group (Ser and Thr) is also associated with class II enzymes. Together with the class II aaRSs of the early group, this makes an entire class II zone for Gly, Ala, Pro, Ser, and Thr (Figure 2, in orange). The next zone (to the right in green) is covered by class I aaRSs and includes the amino acids from the middle and part of the AU-rich group (Cys, Trp, Leu, Val, and Ile). The last codons of the AU-rich group are mixed for both aaRS classes. The zonal distribution of aaRSs overlapping the chronological order of assignment of codons suggests specific timing and interdependence of the initial association of proto-aaRSs to their cognate amino acids. The initial period employed predecessors of class II enzymes and lasted until the assignment of Thr from the middle group. The next period began with class I Cys and Trp development until Leu, Val, and Ile codons were coded. From that moment, both classes of aaRSs started to be used in the same chronological period. 

There are also two other interesting coincidences found in Figure 2: 1. the stop codon UGA marks around the boundary between class II and class I zones, and 2. Arg class I aaRS stays alone, preceding the first class II zone. Some obvious questions then arise: how does the phylogenetic trees of the aaRS evolution correlate with the zonal distribution in Figure 2? How can we explain that Arg possesses class I aaRS since most likely class II originated first? What was the function of the stop codon, just between class II and class I zones? Is the appearance of stop codons twice indicative of an important event in the evolution between class II and class I formation?

## 7. Aminoacyl-tRNA Synthetases Phylogenetic Tree Correlate with the Zonal Distribution

An evolutionary analysis published by O’Donoghue and Luthey-Schulten split aaRS into superclusters based on structural and sequence alignments. For class II aaRSs, these are (ThrRS, ProRS, SerRS) and (LysRS, AspRS, AsnRS) [60]. In a recent study by Burton and coworkers, evolutionary distances were built largely based on *Pyrococcus furiosus* aaRS enzymes with sequence and structure-matching tools utilizing the Phyre2 algorithm [24]. The homology groups found by the Phyre2 algorithm generally agree with the O’Donoghue and Luthey-Schulten data. For class II aaRSs, they find that GlyRS-IIA is next to ProRS-IIA; close to those two are SerRS-IIA, ThrRS-IIA and HisRS-IIA making a separate cluster (in abbreviations like IA or IIA, I stands for class I, II for class II and letters—A–E—for different structural subclasses) [24]. This cluster of five aaRSs is in agreement with our chronologically earliest class II zone formed by Gly, Pro, Ser, and Thr. The second class II zone could begin with the formation of HisRS out of the first class II aaRSs. The next clusters of class II aaRSs are also in agreement with our zonal distribution, where AspRS-IIB, AsnRS-IIB, and LysRS-IIB are evolutionary close. PheRS-IIC is distant from the previous clusters suggesting a later origin [24]. The second class II aaRSs zone contains the enzymes for Asp, Asn, Lys, and Phe, and it is in agreement with Burton’s lab data showing evolutionary kinship. 

The origin of class II aaRS for Cys is more complicated. In some prokaryotic organisms, Cys-specific tRNA (tRNA-Cys) is recognized by a noncanonical class II aaRS (SepRS) charging it with O-phosphoserine (Sep). Then the SH group is added to Sep by SepCysS synthase converting Sep-tRNA to Cys-tRNA-Cys [61]. In that particular case, we can simply assume that class II aaRS for Cys is closely linked with the evolution of SerRS. 

How does class I aaRS zones (Figure 2) agree with existing phylogenetic trees? O’Donoghue and Luthey-Schulten split aaRS into the following superclusters given in parentheses: (GluRS, GlnRS), (TrpRS, TyrRS), and (ValRS, IleRs, LeuRS, MetRS) [60]. We find Val, Ile, Leu, and Met in the same class I zone. Met is far at the end, which is consistent being assigned after Val, Ile, Leu. In addition, Glu and Gln are in the same region of the AU-rich group, making an extension of the class I zone from the Val, Ile, Leu set. The class I data of Burton and colleagues [24] are in line with the zonal distribution of aaRSs as well. LeuRS-IA, IleRS-IA, ValRS-IA, and MetRS-IA are shown to be evolutionarily close and belonging to the same cluster. Close to it are ArgRS-ID and CysRS-IB, which in our order (Figure 1) chronologically precede the Leu, Ile, Val, and Met codons. The most related to CysRS-IB are MetRS-IA, LeuRS-IA, ArgRS-ID, ValRS-IA, and IleRS-IA. Taking Burton and coauthors data and our chronological order, we see no contradiction with the possibility that protoforms of class I ArgRS and CysRS were the sources for the development of LeuRS, IleRS, ValRS, and MetRS. A different and quite likely possibility is that these two branches have a common ancestor. Another related group in the data [24] includes LysRS-IE, GluRS-IB, and GlnRS-IB, where LysRS-IE has some similarities with ArgRS-ID and CysRS-IB. However, GluRS-IB and GlnRS-IB are mostly similar only to LysRS-IE and between themselves. As the authors suggested, GlnRS-IB originated as a modification of GluRS-IB, and the latter in turn likely originated from LysRS-IE. The entire cluster is located in the AU-rich region and therefore chronologically close to each other, indicating a good correlation between the data generated by Burton and coauthors and our zonal distribution in Figure 2.

On the contrary, the cluster consisting of TyrRS-IC and TrpRS-IC [24] does not agree with the order we present in Figure 2. Trp codon is far into the middle group, while Tyr codons are positioned into the AU-rich group, much closer to Glu and Gln than to Trp. In addition to these data, other independent studies [62,63] clearly suggest that TyrRS is older than TrpRS, an exact opposite to the order in Figure 2. While the data suggest TyrRS is older than TrpRS, in our GC/AU order, it is the opposite. How can this discrepancy be explained? The fact that Trp is coded only by one codon (UGG) and the fact that this codon is almost identical to the first stop codon UGA, the only difference being the third base, a possible explanation emerges. Most likely, the Trp codon UGG was initially a stop codon. Later, this particular stop codon lost importance due to the appearance of new AU-rich stop codons, and it was reassigned to TrpRS in a late event of code development. The reassignment of codons, in general, is not an unusual event [64]. In fact, stop codon reassignment happened several times during different stages of evolution, and these became codons for canonical or noncanonical amino acids. Even contemporary stop codon UGA is reassigned to Trp in some bacteria [64]. The Trp/UGG codon reassignment possibility clearly shows that the apparent discrepancy, in fact, confirms our model. If a primitive organism with a UGG stop codon is found, it will be an additional confirmation of our hypothesis. In summary, evolutionary analyzes of aaRSs in earlier studies are in good agreement with the zonal distribution of aaRSs based on codon GC-richness (Figure 2). The evolutionarily close aaRSs are also close within the zonal distribution. 

Why does ArgRS of class I stay oddly at the beginning of GC-rich codons while all remaining GC-rich codons correspond to class II aaRSs? To explain this phenomenon, we need to revisit some of the hypotheses attempting to explain the origin of life.

## 8. Models for the Origin of Life 

Currently, many hypotheses are attempting to explain the abiotic origin of life [65]. We can summarize them in at least three major types based on what “appeared first” suggestions: metabolism-first, RNA world, and RNA–peptide world. It is almost certain that prebiotic synthesis produced a huge number of chemical species with their corresponding interactions [18]; therefore, the idea for a system with a few relatively “pure” building components like RNA, amino acids, and lipids is extremely unlikely. The metabolism-first concepts suggest that the process of prebiotic synthesis and formation of the first living complexes are the same type of events, and they took place before the emergence of genetic information [65]. These concepts organize a living structure from a variety of self-assembling polymers, catalytic metabolic networks, and metabolism reactions [66,67]. Metabolism-first hypotheses are very promising in the description of suitable prebiotic syntheses. However, in order to explain the specific codon formation where a group of amino acids is associated with a specific GC/AU codon ratio, we need to look for hypotheses where RNA and amino acids play the major organizing role. There are two alternative concepts that propose how RNA-dependent Darwinian evolution and life were established: the RNA world hypothesis and the RNA–peptide world hypotheses. The prebiotic RNA synthesis has proven to be challenging; even so, the most probable prebiotic synthesis of nucleotides has been described [51,68]. In addition, the synthesis of pyrimidine nucleosides in one-pot reactions was demonstrated utilizing wet–dry cycles [69,70]. These data suggest that the existence of short prebiotic RNA or RNA-like nucleic acids is a reasonable assumption.

The RNA world hypothesis is based on the ability of RNA to be both a carrier of information and to possess enzymatic activities. It proposes an initial period in which self-sustained entities were composed of RNA only [71,72]. The RNA world, however, requires a polymer transition from RNA only to RNA/peptide/protein complex with de novo formation of proto-translation machinery. In this scenario, the codons are formed independently from their corresponding amino acids, i.e., codon formation and the translation are two separate processes in their origin. The formation of a network of functional ribozymes before codon formation requires a relatively well-established representation of all four nucleotides. It is very difficult to consolidate the fact that some GC-rich codons are specifically related (via codon assignment) to specific amino acids like Arg, Pro, Gly, and Ala since the ribozymes are already selected towards four-nucleotide functionality. 

The alternative for the origin of life is the RNA–peptide world hypotheses. The RNA–peptide world hypotheses are rooted in the mutual benefit of short RNAs and short peptides in the stable complex capable of Darwinian evolution [16,25,73,74,75,76,77]. This avoids the need for polymer transition postulating the existence of primitive translation from the very beginning. In the RNA world and RNA–peptide world models, each amino acid received its codon sequence for different reasons. In the RNA world hypothesis, the codons are formed without the need for translation and peptide formation. Later, during the hypothetical polymer transition ribozymes capable of aminoacylation would fit the first amino acids with their cognate codons, the amino acid playing a role to stabilize or modify the activity of the ribozyme. This scenario does not explain the evolutionary need to develop aminoacyl tRNA synthetases as a totally different way of adapting the amino acid to its cognate codon, confirming again that RNA world is unlikely to explain the assignment of Arg, Pro, Gly, and Ala to particular GC-rich codons. 

Due to the need for RNA/peptide interactions and utilization of the available nucleotide sequence for codon formation, the RNA–peptide world concept explains better the GC-rich association of codons. We assumed that cyclical changes in temperature and/or wet/dry cycles in the prebiotic earth conditions would cause short fragments of random RNAs to undergo hybridization/denaturation cycles. Some of these RNA molecules would have been aminoacylated, and hybridization would bring the RNA termini and the attached amino acids in close proximity. This would, in turn, facilitate peptide bond formation between the closely spaced amino acids, leading to the production of short peptides (termed hybridization dependent peptides—HDPs). The sequence of the HDPs initially would be noncoded and random, reflecting mostly the distribution of amino acids in the environment (showed in Table 1). It has been shown that short peptides could be selected and become nonrandom following cycles of selection, generating self-assembling structures that may create a dynamic combinatorial peptide pool [78]. This experiment suggests the possibility of a variety of prebiotic peptide activities from which a specific RNA binding peptide should emerge. In contrast to peptides synthesized by direct peptide bond formation, HDPs are a kind of peptides that are synthesized by the RNA-mediated hybridization process. This creates a new set of circumstances where RNA-based Darwinian selection could emerge.

**Figure 3:**
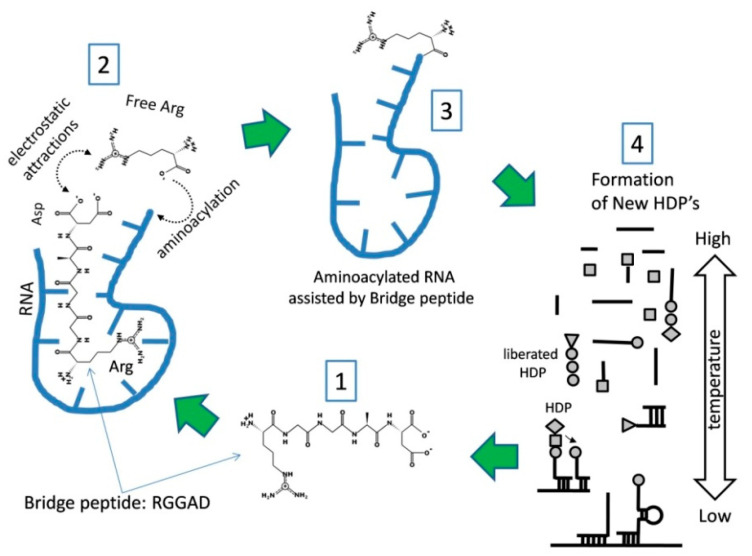
Suggested mechanism for the establishment of Darwinian evolution by BP-mediated aminoacylation (see the text for details).

Our hypothesis also postulates that there is a reasonable chance that a kind of HDP would emerge, which by virtue of being able to bind (or to attract) a specific amino acid (or a group of similar amino acids) and a specific RNA sequence, could lead to aminoacylation that is not random. We call these HDPs “bridge peptides” (BPs) and propose that they (via biased aminoacylation) initiated a positive loop of RNA–peptide complex stabilization and Darwinian evolution (Figure 3) [16]. As an outcome of our concept, Darwinian evolution emerged because of the importance of initial proto-code for RNA–peptide stability. Thus, the genetic code emerged as a consequence of the major property of the BPs to increases the probability of interaction between a specific amino acid and a specific RNA sequence facilitating its aminoacylation (Figure 3). The “successful” combinations would place a given amino acid into a “proper” place in the peptide sequence more frequently than it would have when happening randomly. In our scenario, the evolution of the genetic code is a continuous transition from the prebiotic “chemical code” (with most amino acids being incorporated into HDPs in proportion to their abundance) to an active process of specific aminoacylation, initially driven by BPs and later by evolved proto-aaRS forms. The RNA–peptide world is also in agreement with the coevolution of tRNA, ribosome (LSU and SSU), mRNA, and the genetic code [16]. Those three types of RNAs should act together, not alone, in order to fit and develop better specificity of codon/anticodon detection. Clear evidence for the coevolution of tRNA and rRNA agrees with analyses demonstrating that the 16S and 23S rRNA, and especially the PTC contain sequences similar to those of tRNA [13,15,79,80].

The BP assisted aminoacylation mechanism (Figure 3) permits codon assignment for low abundance amino acids to precede that of a highly abundant one, even if the two had a similar impact on stability. The early “fixation” of a rare (but important, stability-wise) amino acid would be very beneficial, as it is much more probable to obtain the high abundance amino acid in the “correct” position simply by chance. For this reason, we assume that a relatively abundant amino acid might have played a substantial role in peptide stability for a long time without the need to be associated with a specific codon. Thus, it would be misleading if we consider the abundance criteria as the major force in the codon assignment. An example of this type of selection may involve Asp (which is more abundant) and Arg (less abundant), which together, could have played an essential role in BP function (Figure 3). Arg received its codons early due to its low abundance and high importance. On the contrary, the highly abundant Asp could more frequently occupy its “correct” location in the BP just by chance, without the need of a specific codon. This explains the oddity of class I ArgRS being in the GC-rich group (Figure 2), as Arg needed to be coded early into BP formation. 

The early Arg codon assignment correlates with other facts. Class II aaRSs contain conservative motifs “1”, “2” and “3”. Of these “2”, “2B” and “3” possess two highly conserved Arg residues [55,58,81,82]. Class I aaRSs possess highly conserved HIGH and KMSKS motifs [55,81]. They do not contain any conservative Arg but have a preference for hydrophobic amino acids like Leu, Val, or Ile as part of backbone brackets at position 1361 [55]. Proto-class II aaRSs utilized coded Arg as it is suggested from the BP hypothesis before class I formation. Class I evolved later and took an alternative way, utilizing “backbone brackets” with Leu, Val, and Ile, and the corresponding proto-aaRS appeared after the initial class II zone (Figure 2). A logical question arises: How was Arg coded and utilized for proto-class II aaRSs, before having a cognate class I aaRS? In our view, the answer is hidden into the electrostatic interactions of Arg compared to those of other early amino acids. These electrostatic interactions had very low specificity, but good enough to perform primitive translation without the need for a more complicated folded motif (Figure 3). At the same time, the necessary 3D motifs were developed for proto-class II aaRSs to hold nonpolar early amino acids. Later, after establishing Darwinian evolution by the BP, protein development went from very short peptides to longer peptides, which became more specialized with folded 3D shape [31,53,83]. The evolution of the genetic code proceeded from just a single coded amino acid, based on electrostatic interactions to a set of coded amino acids for class II with GC-rich codons and later for AU-rich codons for class I aaRSs.

What could the structure and mechanism of the BP that allows it to recruit Arg to GC-rich RNA be? How did the function of the BP lead to the formation of Darwinian evolution? Although the detailed mechanism of BP-mediated aminoacylation needs experimental confirmation, it has been suggested before [16] and it is shown here with extra details (Figure 3).

The mechanism of BP action is similar to the idea for molecular imprinting put forward by Wulff and Sahran back in 1972. This concept is well developed nowadays, with many applications [84]. Here however, in primitive form, the BP interaction with GC-rich sites on RNA generates a higher probability for the synthesis of more BPs in a positive feedback loop. A BP (Figure 3, Step 1) is initially synthesized by a hybridization-dependent mechanism (see Step 4) by nonspecifically aminoacylated RNAs. The sequence here suggested as Arg-Gly-Gly-Ala-Asp (RGGAD) may vary, and similar sequences may function in a similar way, but to function as BP at least Arg (R) and probably Asp (D) should be present. (Figure 3, Step 2) The BP interacts with RNA because Arg is part of its sequence. The interaction between the BP and RNA occurs predominantly at GC-rich sequences. On the opposite side of the BP, Asp does not interact with RNA, but a mutual attraction between its negatively charged carboxylate ion and positively charged guanidinium group of free Arg occurs. The induced proximity “bridging” facilitates RNA aminoacylation on the ribose -OH group of the last nucleotide at the 3′ end of the RNA. The entire RNA–peptide complex is more stable and circulates longer in the environment, which could also be compartmentalized by rock microchambers, lipid layers or both. At that initial level of complexity, RNA may vary in size and possess GC-sequence variations. The BP aminoacylated RNA will be liberated by a thermal cycle (Figure 3, Step 3) and will be involved in the next round of hybridization dependent peptide (HDP) formation in Step 4. The process of HDP formation (Figure 3, Step 4) described earlier [16] is driven by random, as well as BP assisted aminoacylated short RNAs (from Step 3). Hybridization will occur at low temperatures and bring the amino acids in close proximity to form peptide bonds. The short RNAs are presented as lines, and the amino acids are represented by a circle, triangle, diamond, or squire shapes (Figure 3, Step 4). This process will produce new BPs, which will perpetuate new rounds of synthesis (Steps 2, 3, and 4). Due to the increased stability, the odds of de novo formation of BPs are increased with every cycle resulting in the more frequent introduction of BP-assisted aminoacylated RNA in Step 4. The BP “links” predominantly GC-rich RNA with aminoacylation of free Arg. This will increase its presence in peptides as Arg-aminoacylated RNA will more often enter in Step 4. On the contrary, the amino acids incorporated without bridging will continue to do so solely driven by their abundance (the chemical code) as there is no mechanism to impose specificity. BP action introduces the transfer of information from the GC-rich RNA sequence to Arg. BP formation determines specific positioning of Arg relatively to the other amino acids into the newly formed peptides, which “seeds” formation of the first proto-codon. The process (Step 1 to 4) brings heredity via the primitive transfer of sequence information from GC-rich nucleotides to newly formed BPs by a process that equals primitive translation. This BP-dependent process generates a more stable complex in comparison to other types of newly formed RNA–HDPs due to the increased probability of the BP to repeat itself. As a result, this mechanism (of steps 1, 2, 3, and 4) is more “successful” and will diversify, creating more complex peptides with new codons to increase the stability of the system.

## 9. Simplified Chronological Model in the Transition from Bridge Peptide to aaRSs 

**Figure 4:**
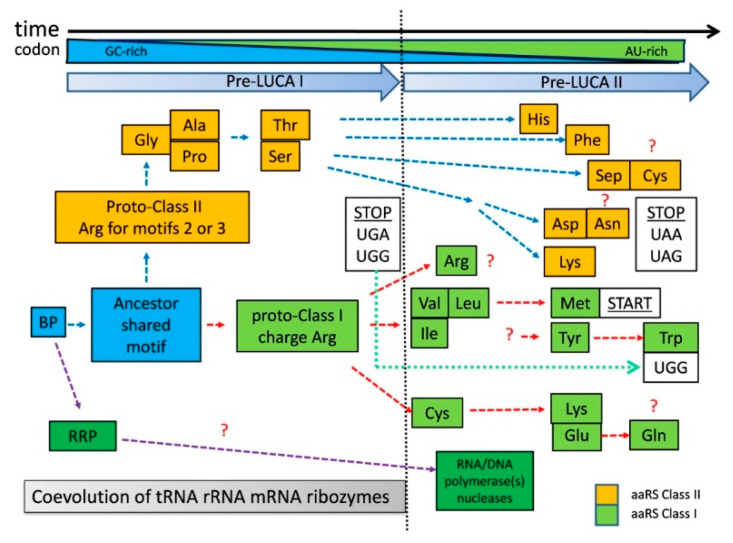
A simplified chronological model of codon assignment during pre-LUCA period. The model is based on GC zonal distribution (Figure 2) combined with published phylogenetic trees of aaRSs. The assignment of codons from early to late is presented horizontally from left to right. The chronological order within each group (e.g., His, Phe, Asp and Lys) may not be exact due to the limitations of the approach. The question marks in red “?” indicates undefined origin. Darwinian evolution initiated as described in Figure 3 favors development of more complicated forms (i.e., proto-aaRS) able to specifically interact with additional amino acids, thus assigning new codons. Based on Figure 2, we suggest two major periods of code development along with aaRS formation. Pre-LUCA I is the first period where only class II proto-aaRS existed with mostly GC-rich RNA and charge Gly, Ala, Pro, Thr and Ser by protoforms of GlyRS, AlaRS, ThrRS, ProRS and SerRS. During pre-LUCA I, there were only short peptides with few motifs and limited functionality, therefore, for some of the essential functions of ribozymes would be selected. The pre-LUCA II period was established after the fidelity of the RNA-replication peptide was enhanced to incorporate efficiently A and U bases during the first enzymatic RNA replication. From that time on, the nucleotide “landscape” increased along with the combinatorial ability for new codon assignment. Pre-LUCA II began with the formation of the first protoclass I aaRS and codons for Leu, Val and Ile (LeuRS, ValRS and IleRs) and continued with the evolution of both class I and class II. (see the text for details).

The zonal distribution of aaRSs (Figure 2) relative to the chronological order of codon assignment should indicate the chronological order of proto-aaRS development. The evolutionary phylogenetic trees of aaRSs [24,60] provide essential information about the evolutionary similarity between different aaRS. Based on our chronological order of codon assignment and the phylogenetic data, the evolution towards LUCA occurred in at least two major periods (Figure 4). The first one (pre-LUCA I) is the development of proto-class II aaRSs only using predominantly GC-rich codons. In the second one (pre-LUCA II) class I aaRSs were developed and continued the development of class II aaRSs. These two periods of LUCA are separated by the appearance of first stop codons suggesting the evolutionary need for “in-frame” translation at the end of pre-LUCA I. Pre-LUCA I contained only proto-forms of class II aaRSs for coded Gly, Ala, Pro, Ser, and Thr. As explained in Figure 3, the codon formation began during the transition from noncoded HDPs to coded HDPs. The first GC-rich codons formed around a nucleotide, which would emerge later as the second (middle) base in the tri-nucleotide codon/anticodon recognition. Until the end of pre-LUCA I, the three-nucleotide codon system would be established, mostly utilizing GC nucleotides. All coded peptides during this period were relatively short with one or maybe two simple motifs serving mostly aminoacylation, RNA replication, and RNA stabilization; all evolved from the BP containing Arg and perhaps noncoded Asp.

During this initial period, there is no need for advanced forms of proto-aaRS to recognize Arg, because the BP and its variants interacted with Arg mostly based on electrostatic attraction. Therefore, Arg aminoacylation would be performed mostly from closely related to the initial BP peptides, using Arg (Figure 3). On the contrary, the necessary Asp (to attract Arg, Mg^2+^, Fe^2+^ or Mn^2+^) would be aminoacylated by chance (no code necessary) due to its relative abundance. The “chemical code” still played a role in this period [16] (Table 1). This is the reason why class I aaRS would not be developed until the next pre-LUCA II period (Figure 4). In contrast, Gly, Ala, Pro, Ser, and Thr, being only polar or neutral amino acids, cannot be charged on RNAs/incorporated “easily” and would require the folded motif of the future class II aaRSs in order to be recognized. Protoclass II utilizes Arg residues in its motifs “2” and “3” for the ATP binding catalytic pocket (forming “Arg tweezers”). This is in agreement with the existence of coded Arg during those very early steps. During this period, the stop codons UGA and UGG are established, securing the fixed stop and “in-frame” translation of the first short peptide motifs. 

A very intriguing suggestion has been made by Burton and collaborators, showing that class II and class I aaRS are, in fact, homologs [24]. The authors found shared β-sheets that organize the Zn fingers, suggesting a common origin. According to them, the formation of class I (especially for IleRS-IA and ValRS-IA) came from an initial proto variant GlyRS-IIA, where the N-terminus extended and refolded as the first class I [24]. The main hypothesis of these authors is built on the assumption that GlyRS-IIA was the very first aaRS, and Gly was the very first amino acid to be coded. From the perspective of BP-dependent Darwinian evolution and codon formation, it is logical to assume a shared BP-derived folded peptide serving as an ancestral form (missing link) of both classes of aaRSs (Figure 4). In that case, reasonably, GlyRS-IIA would have shared Zn-finger with IleRS-IA and ValRS-IA. The β-sheet(s) are highly advanced motifs for the time of BP and its close evolutionary forms to exist. In addition, according to our model, it is quite acceptable that GlyRS-IIA was the first folded aaRS longer peptide. However, this does not mean that it was the first to perform specific aminoacylation. During this initial period, Arg aminoacylation would depend mostly on its electrostatic charge and RNA interactions (Figure 3), not on a folded motif.

The proposed development (Figure 4) of pre-LUCA I with a limited number of coded amino acids and primitive class II, offers a possible answer to another enigmatic question in the evolution of the translational apparatus. It seems that the classical L-shape tRNA cloverleaf fold in an almost complete form evolved quite early in pre-LUCA time, before the establishment of the entire genetic code [23,24,75]. A most likely scenario for the evolution of cloverleaf fold L-shaped tRNA was published by Burton and coworkers [24,79,85]. First, the primordial tRNA developed from contiguous GC-rich repeats like (GCG)_3_ and (TAGCC)_4_ to form the acceptor stem and D-loop. Second, the anticodon and T loops took shape from the 17nt microhelix sequence [23,24,79]. In addition, the SSU and LSU of the ribosome RNA also should have had a relatively “fast” initial co-development in order to “fit” with tRNA. RNA world scenario used this argument to suggest the formation of the genetic code before the appearance of translation in an ancient ribozymes-mediated operational code [1]. This concept is incompatible with the RNA–peptide type hypotheses, where the translation and the formation of genetic code are two mutual and inseparable processes. As our model shows, there is no need to separate those two fundamental processes (codon formation and translation) because the translation system was developed only on the level of few amino acids during pre-LUCA I, and tRNA and rRNAs were fitted relatively quickly due to the simple code and a small number of coded amino acids during that time. Once the translation machinery obtained its functional shape, i.e., an almost complete tRNA L-shape and an almost complete initial core of functional SSU and LSU of the ribosome, it paved the way to the next level of codon expansion where novel forms of aaRS and new amino acids were assigned. Almost complete tRNA shape and rRNA surely required AU nucleotides, which agree with the notion that GC-rich RNA was selected for codons assignment. Primordial RNA, indeed, may be GC-rich, but not A or U free. In that case, the fewer A and U nucleotides would be more important for tRNA, rRNA or the new functional ribozymes selected at that time, instead to code the few useful amino acids in pre-LUCA I. The very beginning of pre-LUCA I represents a true RNA–peptide world where only a few short peptides and a few simple folded motifs with limited functions did exist. Due to the lack of long peptides to cover some of the important functions at the time, ribozymes would be naturally selected (e.g., RNase P). A good example of selection during pre-LUCA I is the ribosomal LSU, which is a ribozyme capable of peptidyl transfer. Few selected peptides, however, happened to be more suitable to facilitate functions like aminoacylation, RNA replication (Figure 4), and maybe some of the first metabolic reactions. RNA replication peptide (RRP) and its evolved forms should have facilitated the incorporation of A and U ribonucleotides, creating the possibility for new combinations of codons. In addition, already L-shaped tRNA, ribosomal SSU and LSU would facilitate the beginning of the next evolutionary step (pre-LUCA II) where new and modified tRNA/aaRS pairs would evolve. 

Pre-LUCA II should have begun (Figure 4) when advanced forms of the BP as protoforms of class I (initially facilitating aminoacylation of Arg only) began to recognize Val, Ile, and Leu utilizing U/A as the middle base. Later, the same aaRSs evolved to recognize Met as well. Most likely, the first variant of the proto-aaRS for Ile, Val, Leu, and Met was not able to recognize them individually since they are very similar to one another and have similar codons. It is very appealing to assume that class I aaRS adenosine phosphate-binding domain utilized two highly conserved backbone hydrogen bonds (backbone brackets), showing a preference for hydrophobic amino acids like Leu, Val, or Ile [55]. These are exactly the amino acids charged by class I, which should have become encoded at the beginning of pre-LUCA II. There is another peculiar correlation. Recently, Lupas and coworkers described the antiquity of the KH motif traced back to LUCA time [54]. KH motif utilizes (I/L/V)IGxxGxx(I/L/V), a conservative pattern that interacts with single-stranded nucleic acids; therefore, its function would be relevant to RNA–peptide interactions. Today, the KH motif is found in a variety of proteins with diverse functions (i.e., prokaryotic RNase G/E/Y, PNPase, RRP4) [54]. It is very intriguing that, again, all conservative Ile, Leu, and Val (Figure 4) were the first assigned codons at the beginning of pre-LUCA II. The origin of any folded motifs should have followed the initiation of Darwinian evolution. Therefore, KH type motif(s) or backbone brackets have to originate after the first stabilized structure with only short (e.g., 3-5 amino acids) unfolded peptide(s) (explained in Figure 3). The beginning of pre-LUCA II (Figure 4) is a suitable time for the emergence of class I precursor for IleRS, LeuRS and ValRS because the encoding of Ile, Leu and Val would have allowed more advanced and longer backbone brackets for ATP binding or advanced more specific RNA binding using evolved folded motifs. UGG, the former stop codon from the pre-LUCA I period was reassigned to Trp as a late amino acid. Additional stop codons (UAA and UAG) were required, which, together with the first stop codon UGA, became standard stop codons. For already longer proteins, with fixed start and end for “in-frame” translation, the common start codon was established out of Met (AUG) codon. The evolutionary process of proto-aaRS continued its codon and amino acid optimization towards more mature “final” variants due to processes such as mutagenesis, horizontal transfer of genes, and selection after LUCA formation. Based on our arguments, it is very difficult to determine when a true proto-ArgRS was established. Initially, Arg was charged only based on electrostatic interactions with GC-rich RNA during pre-LUCA I. However, at some point during pre-LUCA II, the folded proto-ArgRS emerged directly from proto-class I aaRS. It is quite possible the last two codons for Arg (AGA and AGG) were assigned during pre-LUCA II. Figure 4 represents only a simplified global evolutionary model, and more details should be added in the future using more sophisticated methods of research.

## 10. Conclusions

In the current paper, we describe many correlations evident in the universal genetic code: The most GC-rich codons encode amino acids, which likely contributed the most to RNA–peptide interactions. According to the RNA–peptide world hypothesis, the initial amino acids should be incorporated into peptides capable of stabilizing the complex, which initiated Darwinian evolution. The most straight-forward condition for any amino acid to be associated with a given codon sequence is the vacancy of nucleotide combinations at the time when the amino acid would be essential for survival. Since the amino acids with GC-rich codons could form peptides suitable for RNA interactions, we could hypothesize that the earliest RNAs were predominantly GC-rich. This conclusion is supported by the difficulty of incorporating A and U in experiments aiming at nonenzymatic template-directed in vitro RNA replication, as well as by the composition of the evolutionary oldest segments of tRNA and rRNA. This leads us to a straightforward conclusion: the amino acids that possess GC-rich codons were the very first to be included in the initial living complex. In addition, the BP concept we have proposed previously [16], requires the incorporation of these amino acids, as they stabilize RNA the most.

When we arranged codons by their GC-richness (Figure 1), we found another correlation—the amino acids outside of the GC-rich group tend to promote protein folding. This is exactly what would be expected after the development of more complex and longer proteins containing folded motifs and finally proteins with a fixed start and end [31]. If the evolution of the genetic code went from GC-rich to AU-rich codons (at least on a global scale), the evolution of the corresponding aaRSs also should have followed a similar pattern. By overlaying the corresponding aaRS over the nucleotide composition order of codons (Figure 1), the zonal distribution of class I and class II emerged (Figure 2). The distribution suggests evolutionary periods of co-evolution of aaRS together with codon assignment. Phylogenetic data of aaRSs clearly confirms that the evolutionary closely related groups also fall in the same zonal groups (Figure 2). Together, phylogenetic data and our aaRS zonal groups (Figure 2) suggest the timeline of codon assignment and the evolution of aaRSs during the pre-LUCA time. We propose a chronological model (Figure 4) of two major periods of codon/aaRS development: During pre-LUCA I only class II proto-aaRS, associated with GC-rich codons, developed. In pre-LUCA II, when AU-containing RNA became available, the new codon landscape allowed for the establishment of the codons for the rest of amino acids and aaRS, paving the way to the evolution of developed LUCA containing an estimated ~100 proteins and approximately 300 genes [86]. There are at least four models [86] of how LUCA gave rise to all life domains, which complicate the chronological description of the very last coded amino acids (canonical and noncanonical).

Taken together, the correlations (Figure 2) we discussed, suggest a simple model (Figure 4) with a lot of explanatory power. It supports the RNA–peptide world hypothesis, in which early BPs (Figure 3) initiated Darwinian evolution with largely GC-rich RNA–peptide interactions, followed by the evolution of the entire universal genetic code during the pre-LUCA period. The BP concept and the first steps of RNA–peptide interactions (Figure 3) could be experimentally proved or disproved. It is quite feasible to simulate primordial Earth conditions in the laboratory. In these experiments, we expect HDPs formation and Arg containing peptides to be sufficiently stable with GC-rich RNA showing properties of primitive translation.

## Figures and Tables

**Table 1:** Abundance of prebiotic amino acids (chemical code) relative to glycine (Gly). Data modified from [17]. (a) Relative abundance from experiments using simulations in environments such as atmospheres and hydrothermal vents. (b) Relative abundance experiments using interstellar medium simulations and meteorite analyses.

(a)		(b)	
Amino Acid(s)	Abundance	Amino Acid(s)	Abundance
Gly	1.0	Gly	1.0
Ala	2.00–0.30	Ala	0.50–1.50
Ile, Val	0.30–0.05	Ser, Glu	0.50–1.00
Ser, Glu, Pro, Asp	0.15–0.02	Asp	0.2–0.80
Leu, Phe, Thr, Cys, Met, Arg, Lys, His	0.05–0.01	Val	0.10–0.30
